# Supplementary material for: Zasp52’s differentially expressed intrinsically disordered region confers thin filament stability at the Z-disc
Source: eLife. 2026 Aug 27;15:RP111101. doi: 10.7554/eLife.111101 (PMC13521563; doi:10.7554/eLife.111101)
Supplement: Supplementary file 2 [file elife-111101-fig1-data1.zip › Figure_1-source_data_1-1.pdf]

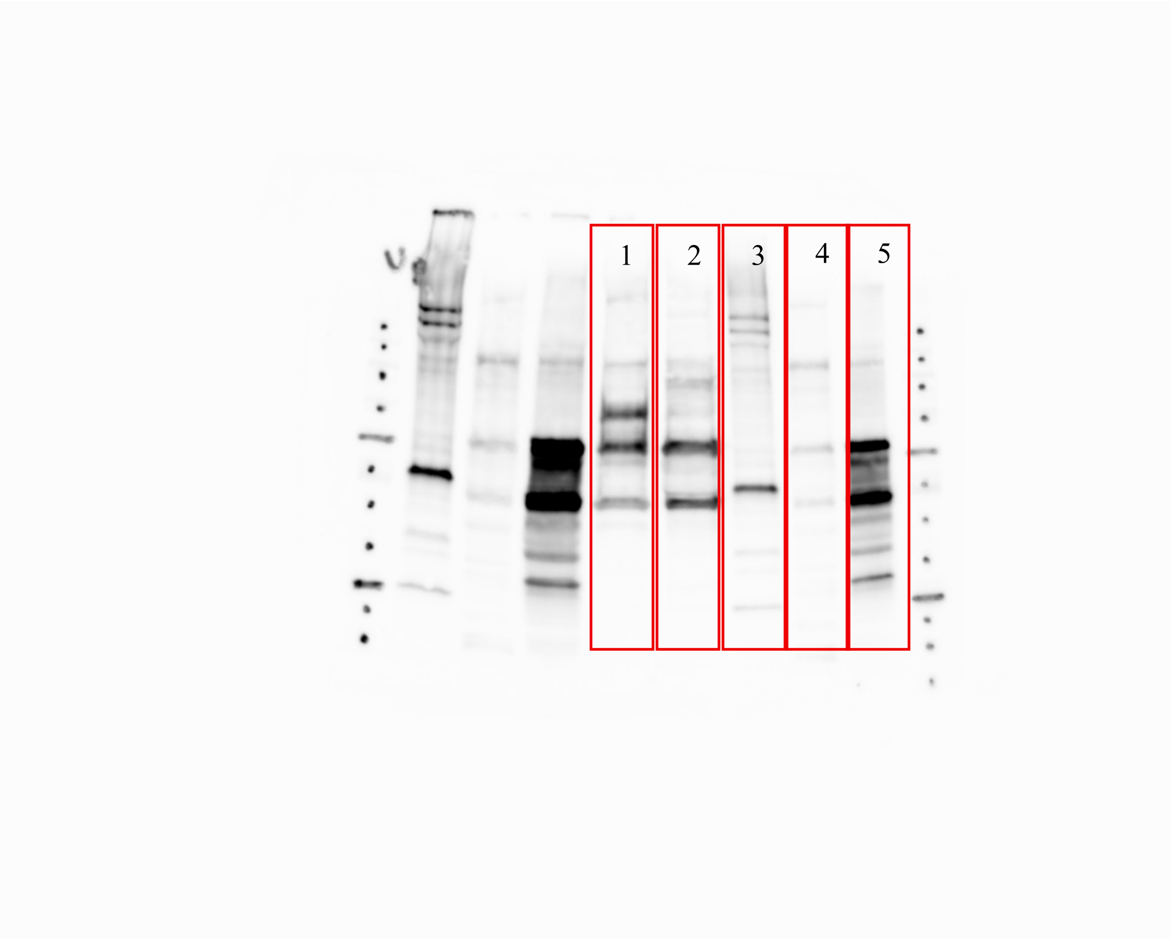

Figure 1B

1: head

2: abdomen (male)

3: IFM

4: ovary

5: larva
