# Supplementary material for: Zasp52’s differentially expressed intrinsically disordered region confers thin filament stability at the Z-disc
Source: eLife. 2026 Aug 27;15:RP111101. doi: 10.7554/eLife.111101 (PMC13521563; doi:10.7554/eLife.111101)
Supplement: Supplementary file 4 [file elife-111101-fig2-data1.zip › Figure_2-source_data_1.pdf]

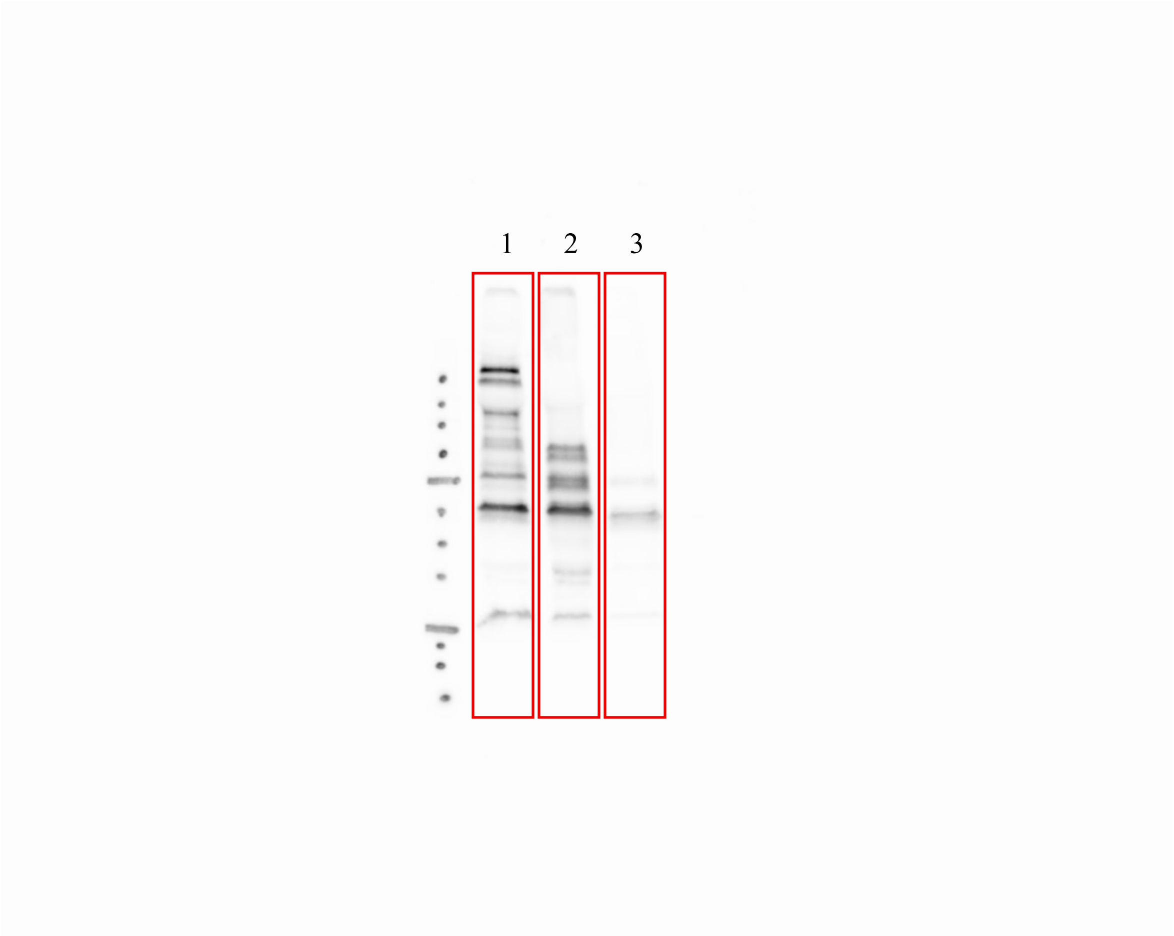

Figure A

1: ctrl

2: *ex15e*

3: MI00979

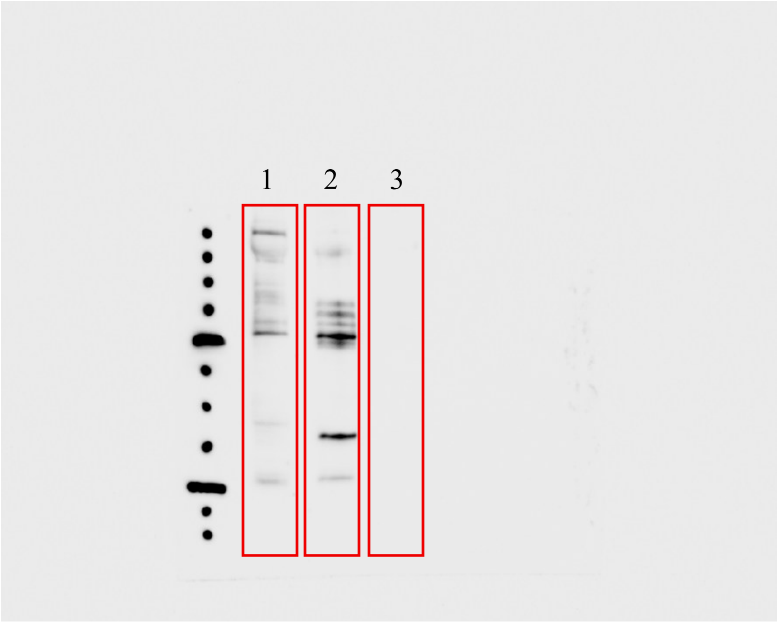

Figure A’

1: ctrl

2: *ex15e*

3: MI00979
