# Supplementary material for: Zasp52’s differentially expressed intrinsically disordered region confers thin filament stability at the Z-disc
Source: eLife. 2026 Aug 27;15:RP111101. doi: 10.7554/eLife.111101 (PMC13521563; doi:10.7554/eLife.111101)
Supplement: Peer review history

# Editor report: eLife Assessment

**DOI:** 10.7554/eLife.111101.3.sa0

**Authors:** Michael Buszczak

**Affiliations:** University of Texas Southwestern Medical Center United States

**Keywords:** Convincing, Important

This study investigates the role of the Z-disc protein Zasp52 in Drosophila flight muscles and provides evidence that an intrinsically disordered region (IDR) helps to stabilize and promote the localization of the protein to the Z-disc. Overall, this represents an **important** study that provides insights into Z-disc function and maintenance. The data are **convincing**, supported by strong genetic evidence and behavioral tests, well-controlled experiments, and detailed statistical analyses. Characterization of a new actin-binding motif mutant and FRAP analysis further support the functional importance of the Zasp52 IDR.

# Referee report: Reviewer #1 (Public review)

**DOI:** 10.7554/eLife.111101.3.sa1

[Editors' note: this version has been assessed by the Reviewing Editor without further input from the original reviewers. The authors have addressed the comments raised in the previous round of review.]

The manuscript by Ho and Schock investigates the role of the Z-disc protein Zasp52 during Drosophila flight muscle development. It was known before, mainly by findings from this group, that Zasp52 is required for normal sarcomere morphogenesis, specifically Z-disc morphogenesis in indirect flight muscles. But the exact molecular mechanism by which Zasp52 contributes, apart from the fact that it is localised there and is somehow involved in multimerization/cross-linking, was not clear. This paper proposes that an intrinsically disordered region (IDR) in Zasp52 is needed for some of its functions, by stabilising Zasp52 localisation at the Z-disc. Specifically, the IDR in Zasp52 is proposed to be required for Z-disc maintenance during the mechanical challenges of flight, while being dispensable for the initial morphogenesis during development. This hypothesis is supported by strong genetic evidence and behavioural tests, deleting Zasp's IDR impairs flight from mid-age onwards, while a block in flight activity lifts the phenotype.

Strengths:

(1) The linker in the alternatively spliced exon 15 of Zasp52 was deleted with a state-of-the-art genetic editing strategy. Surprisingly, flies are homozygous viable, showing that this long part of the Zasp52 protein is not essential for animal survival or sarcomere morphogenesis.

(2) The observed sarcomere phenotypes with age, especially the bending Z-discs, are new and exciting.

(3) The displayed EM images document interesting phenotypes.

(4) Most of the observed phenotypes can be rescued by re-expression of the long Zasp52 isoform, which does contain the IDR region, but not by a shorter one without it, suggesting that IDR is important.

(5) FRAP data measure the local turnover of a short-ZaspGFP and show that this increased in the Zasp mutant lacking the IDR domain, suggesting that Zasp-IDR might stabilise Zasp at the Z-disc.

(6) Interestingly, flight and sarcomere morphology phenotypes can be rescued by preventing the flies from flying, suggesting that they are mechanically induced.

# Referee report: Reviewer #2 (Public review)

**DOI:** 10.7554/eLife.111101.3.sa2

Summary and Strengths:

This in-depth genetic analysis of Zasp52 function in Drosophila indirect flight muscle (IFM) provides an interesting perspective regarding the role of a partially disordered region (IDR) in exon 15e. This exon seems to be exclusively present in IFM and contributes to the prevention of myofibril disintegration during aging, likely due to interactions of this region with Z-disc insertion and/or stability. The addition of an isoform (PR) that lacks exon 15e serves as a nice control to illustrate the necessity of exon 15e in muscle structure and function. Overall, the manuscript is exceptionally well-written, logical, with nicely controlled experiments and detailed statistical analysis that largely support the conclusions drawn by the authors. While exon 15e is clearly involved in preventing muscle degeneration, a solid role for thin filament stability is not clearly shown (as mentioned in the abstract). In addition, which regions/how the proteins of the IDR may contribute are unclear.

# Author comment: Author response

**DOI:** 10.7554/eLife.111101.3.sa3

**Authors:** Nikolai Ho, Frieder Schöck

**Affiliations:** McGill University Montreal Canada; McGill University Montreal Canada

The following is the authors’ response to the original reviews.

Thank you for the helpful comments and criticisms. We provide exciting additional data, in particular a CRISPR actin-binding motif mutant and FRAP analysis of an exon15e-GFP transgene, both further supporting the importance of the IDR in thin filament stability. We believe that these additional experiments provide compelling evidence supporting our conclusion and substantially advance the current limited body of knowledge surrounding the role of IDRs in structural proteins.

**Public Reviews:**

**Reviewer #1 (Public review):**

The manuscript by Ho and Schock investigates the role of the Z-disc protein Zasp52 during Drosophila flight muscle development. It was known before, mainly by findings from this group, that Zasp52 is required for normal sarcomere morphogenesis, specifically Z-disc morphogenesis in indirect flight muscles. But the exact molecular mechanism by which Zasp52 contributes, apart from the fact that it is localised there and is somehow involved in multimerization/cross-linking, was not clear. This paper proposes that an intrinsically disordered region (IDR) in Zasp52 is needed for some of its functions, by stabilising Zasp52 localisation at the Z-disc. Specifically, the IDR in Zasp52 is proposed to be required for Z-disc maintenance during the mechanical challenges of flight, while being dispensable for the initial morphogenesis during development. This hypothesis is supported by strong genetic evidence and behavioural tests, deleting Zasp's IDR impairs flight from mid-age onwards, while a block in flight activity lifts the phenotype.

However, some of the phenotypic analysis, in particular the bending of the sarcomere, likely upon mechanical challenge by muscle contractions, needs more detailed investigations to be fully convincing.

Strengths:

(1) The linker in the alternatively spliced exon 15 of Zasp52 was deleted with a state-of-the-art genetic editing strategy. Surprisingly, flies are homozygous viable, showing that this long part of the Zasp52 protein is not essential for animal survival or sarcomere morphogenesis.

(2) The observed sarcomere phenotypes with age, especially the bending Z-discs, are new and exciting.

(3) The displayed EM images document interesting phenotypes.

(4) Most of the observed phenotypes can be rescued by re-expression of the long Zasp52 isoform, which does contain the IDR region, but not by a shorter one without it, suggesting that IDR is important.

(5) FRAP data measure the local turnover of a short-ZaspGFP and show that this increased in the Zasp mutant lacking the IDR domain, suggesting that Zasp-IDR might stabilise Zasp at the Z-disc.

(6) Interestingly, flight and sarcomere morphology phenotypes can be rescued by preventing the flies from flying, suggesting that they are mechanically induced.

Weaknesses:

(1) The western blot quantifications of Zasp isoform expression are weak. No error bars are indicated in the quantifications; the quantifications appear to be more qualitative than quantitative. According to band intensities, the long Zasp isoforms seem to be less present compared to the shorter ones, even in the flight muscles.

We have now included quantifications with error bars for the Western blots in our resubmission. It is important to keep in mind that the main point in figure 1B is that there are plenty of exon15e-containing isoforms in IFM, in contrast to other tissues with very limited exon15e-containing isoforms. This is confirmed by the analysis of RNA-seq data in figure 1C, and of course, by the flightless phenotype of the exon15e mutant.

(2) The phenotypic analysis of the sarcomere appears somewhat superficial throughout the paper. Only Zasp52 and phalloidin are shown; no other Z-disc or thick filament proteins. At least myosin stainings and overview images are important to better judge the phenotypic variations. Are the variants between individuals or regional in the same muscle?

Our images are representative of the observed phenotypes. Phenotypes are consistently present across all individuals, as reflected in our replicates. Interestingly, they appear not to be randomly interspersed among the sarcomeres but concentrated in certain regions of muscle more than others. Full images are available in the online repository FigShare.

(3) EM images would benefit from better quantification.

We do not believe that EM images can be meaningfully quantified, because of the many selection steps preceding image acquisition.

(4) Other proteins were not analysed with the FRAP-based turnover assay for comparison in wild type and mutant. All Z-proteins might turn over faster in the mutant with the defective Z-disc.

This is the point we are trying to make. The Zasp52 IDR appears to stabilize the Z-disc and is likely involved in fastening a variety of proteins to it.

**Reviewer #2 (Public review):**

Summary and Strengths:

This in-depth genetic analysis of Zasp52 function in Drosophila indirect flight muscle (IFM) provides an interesting perspective regarding the role of a partially disordered region (IDR) in exon 15e. This exon seems to be exclusively present in IFM and contributes to the prevention of myofibril disintegration during aging, likely due to interactions of this region with Z-disc insertion and/or stability. The addition of an isoform (PR) that lacks exon 15e serves as a nice control to illustrate the necessity of exon 15e in muscle structure and function. Overall, the manuscript is exceptionally well-written, logical, with nicely controlled experiments and detailed statistical analysis that largely support the conclusions drawn by the authors. While exon 15e is clearly involved in preventing muscle degeneration, a solid role for thin filament stability is not clearly shown (as mentioned in the abstract). In addition, which regions/how the proteins of the IDR may contribute are unclear.

Weaknesses:

(1) It is not clear in Figure S1A where exon 15e fits within the Zasp52 locus schematic. This is important as a premise of this paper describes this region to be key, and proof from multiple prediction programs would lend more weight to the prediction of the exon being largely disordered. Inclusion of the discussed short linear motifs, comparison with Canoe or LBD3 for similarities and/or an Alphafold structure would help make the authors' point (colorized with known domains).

We added a bar below figure S2A to show the region corresponding to exon 15e. We used three disorder prediction programs and one structure (order) prediction program. The majority of exon15e is completely disordered and of very low confidence score, and thus uninformative to display as an AlphaFold structure. Likewise, IDR’s are very difficult to classify, therefore we cannot say much more than that LDB3, Zasp52, and Canoe contain IDRs, with Zasp52 and Canoe both having a putative actin-binding domain within the IDR. We now provide data on the function of the ABD in this resubmission.

(2) Interesting that immobilization rescues the deterioration phenotypes. The authors should explain in more detail how this was done to avoid dehydration/starvation of the flies.

We provided more details in materials and methods.

(3) There is a lot of discussion about the potential function of the IDR region, specifically a putative actin binding motif or other 'ordered' regions that may contain short linear motifs. It would strengthen the findings to show which of these may be essential for Zasp52 function in the IFM. The ability to bind actin could be tested biochemically, and/or smaller deletions could be made to unequivocally test the role of the ABD vs other predicted motifs using genetics. If some of these regions are more ordered, where do they lie within, and do they form a predicted fold or structure that gives insight into function?

We now provide data on the function of the ABD showing that deleting it has almost no phenotypic defects. That means the IDR is largely/entirely responsible for the observed phenotypes.

**Recommendations for the authors:**

**Reviewer #1 (Recommendations for the authors):**

(1) Western blot in Figure 1B needs proper quantification. A ratio between long and short isoforms in the same muscle type might be informative. Is it known which epitope the antibody recognises? Can a GFP insertion that also labels all isoforms be used as verification? Quantifications are also needed in Figure 2A.

We have added quantifications of all Western blots (Fig. 1B, 2A, and 2A’). The ratio between exon 15e-containing and total Zasp52 in the same muscle type is included in the lowest bar graph in Fig. 1B. The full-length antibody is polyclonal and was raised against Zasp52-PR which contains all ordered domains; the anti-LIM antibody was raised against the last three LIM domains (both are described or referenced in the materials and methods section). Such a GFP insertion cannot exist due to the complex splicing patterns of Zasp52.

(2) The name of the deletion allele could be specifically indicated in Figure 1A below the red bar.

Done.

(3) It would be useful to indicate the order group names in Figure S2B since species names are hard to read.

For the version of record we provided high-resolution images, where species names can be read. *Drosophilids*, *Ephemeroptera* and *Odonata* are indicated.

(4) The inverted spelling of the numbers for the control in Figures 2C and 5H is strange.

Changed to normal spelling.

(5) The bending of the myofibril at the Z-disc is a really interesting phenotype. However, it seems it is not always visible; at least it is visible in many myofibrils shown in Figure 3B, but in none in Figure 3E, same genotype, just different staining. Hence, I wonder if this bending could be force-induced by the cutting of the thorax during tissue preparation. It would be useful to display some overview images to allow the reader to judge the quality of the tissue preparation, indicating from where the high magnification view shown was taken. The same is true for Figure 5.

Overview images are available on FigShare. Note that you can see some “H-zone actin” sarcomeres in Fig. 3B, as well as some mildly bent ones in Fig. 3E. We generally selected images that best demonstrated the phenotype described. Furthermore, neither phenotype is fully penetrant so we cannot expect to see it everywhere. Lastly, it is always possible that phenotypes are affected by preparation, since it is impossible to know what the myofibrils look like in situ. However, all samples were prepared using the same protocol with replicates, and since we see a phenotype in our mutants and not in the control, this indicates that something is different between the two.

(6) The same applies to the visualisation of the "hyper-contracted" phenotype; again, it seems to be an all-or-nothing phenotype in the zoom shown. An overview image should be shown. The zoom in Figure 4E would benefit from displaying phalloidin in a separate channel. Are actin filaments pulled out of the Z-disc? The latter is often seen in non-perfect cuts in wild-type, but the accumulation at the M is curious. It would be informative to locate the ends of the thick filaments in these cases or quantify thick filament lengths; do these invade the Z-discs? This can easily be done by a myosin staining.

Is this a regional effect or does it depend on the individual or on the preparation? I am surprised to also see the "hyper-contraction" in 10% of wild-type 5-day adults.

See previous response where we include overview images. Single-channel images are available; it is visible that actin filaments are not pulled out of the Z-disc. Phenotypes are consistent across individuals as evidenced in our replicates but do tend to be concentrated in certain regions of muscle.

(7) The EM images would benefit from more overview images. At the moment, we only see a single sarcomere from wild type and mutant, with no quantification of the phenotype. Can the authors see the invading thin filaments into the M-band? The disrupted Z-disc phenotypes are impressive. What is the age of the animal shown in Figure 4?

We have a panel displaying several mutant sarcomeres. Due to the selectivity and challenges of the EM preparation process, we do not believe we can perform meaningful statistics on them. It sometimes looks like myosin heads are visible in the H zone which may support the presence of thin filaments in the H zone (Fig. 4B and C). However, the quality of these particular EM images is not high enough to identify thin filaments. All phenotypes shown are from 3-week-old animals.

(8) Is UH-3 GAL4 expressed at the adult stage?

Yes, from 36 h APF into adulthood (Singh et al. 2014). Now mentioned in the results section.

(9) Figure 6 would strongly benefit from a myosin staining. Do thin and thick filament lengths scale? It seems that overlap is reduced in the double hets. How can this be envisioned with Z-disc stability? Is myofibril diameter reduced?

We searched for non-additive differences in myofibril diameter but were unable to detect any.

(10) What is the FRAP turnover rate of a long Zasp-GFP compared to a short one in wild type? A difference would indicate that it is really the IDR domain that keeps Zasp52 longer at the Z-disc, instead of an indirect effect caused by Z-disc morphology

We have newly added FRAP data of a GFP-tagged exon 15e construct which displays much lower turnover. This indicates that the IDR does indeed retain Zasp52 at the Z-disc.

**Reviewer #2 (Recommendations for the authors):**

(1) The total protein stain should also be included if it is used for quantitation in Figures 1B and 2A-A'.

These are available on FigShare.

(2) It is a bit confusing that the Alphafold plot is inversely correlated with the other 3 prediction programs, although this is explained in the legend. Maybe an Alphafold structure would help make the authors' point (colorized with known domains).

The AlphaFold structure is almost entirely low-confidence disordered region except for the structured domains so we do not believe it would be helpful to include.

(3) The title of Figure 8 says 'Certain ex15e defects are rescued by immobilization.' What other defects are not rescued? If true, these should be shown.

There was a full rescue. We deleted the word “certain”

(4) Please include a brief explanation of the spatiotemporal expression of UH3-Gal4.

From 36h APF into adulthood (Singh et al. 2014). Now mentioned in the results section.

(5) Statistics should be added to Figure 8E.

Figure 8E (now 9E) has statistics.

(6) The dark blue color used for integrin staining in Figure S3 is difficult to see. Changing this color may help visualize differences. Also, pointing them out with arrows, etc., will help clarify abnormalities.

We have described these differences in the figure caption. Single-channel images are available for viewing in any color in FigShare.
